# Thermodynamic Properties of Magnesium Oxide and Beryllium Oxide from 298 to 1,200 °K^1^

**DOI:** 10.6028/jres.067A.034

**Published:** 1963-08-01

**Authors:** Andrew C. Victor, Thomas B. Douglas

## Abstract

As a step in developing new standards of high-temperature heat capacity and in determining accurate thermodynamic data for simple substances, the enthalpy (heat content) relative to 273 °K, of high purity fused magnesium oxide, MgO, and of sintered beryllium oxide, BeO, was measured up to 1,173 °K. A Bunsen ice calorimeter and the drop method were used. The two samples of BeO measured had surface-to-volume ratios differing by a factor of 15 or 20, yet agreed with each other closely enough to preclude appreciable error attributable to the considerable surface area. The enthalpies found for MgO are several percent higher than most previously reported values. The values are represented within their uncertainty (estimated to average ± 0.25%) by the following empirical equations^3^ (cal mole^−1^ at *T* °K)

$$\begin{array}{c} \text{MgO}:H_{T}^{{}^{\circ}}-H_{273.15}^{{}^{\circ}}=10.7409T+1.2177\left(10^{-3}\right)T^{2}-2.3183\left(10^{-7}\right)T^{3}+2.26151\left(10^{5}\right)T^{-1}-3847.94.\\ \text{BeO}:H_{T}^{{}^{\circ}}-H_{273.15}^{{}^{\circ}}=11.1084T+7.1245\left(10^{-4}\right)T^{2}+8.40705\left(10^{5}\right)T^{-1}-5.31245\left(10^{7}\right)T^{-2}-5453.21. \end{array}$$

Values of enthalpy, heat capacity, entropy, and Gibbs free-energy function are tabulated from 298.15 to 1,200 °K.

## 1. Introduction

A previous paper [1]^4^ has described the need for heat-capacity standards at temperatures above the range of the present *α*-aluminum oxide (corundum) standard. Enthalpy measurements on thorium dioxide have been presented [1] as a first step in the investigation of new materials for this purpose.

The chemical stability and high melting points of MgO and BeO (3,000 and 2,800 °K, respectively [2]) recommend these materials for consideration as possible heat-capacity standards. Although both compounds have lower melting points than thorium dioxide (3,300 °K [2]), the lower sensitivity of their heat capacities to the influence of common impurities (because of smaller differences in atomic weights) is a decided advantage. The accurate knowledge of the thermodynamic properties of these substances over a large temperature range has added value because of the very frequent occurrence of these materials in high-temperature reactions and installations. The results of enthalpy measurements on fused MgO and on sintered BeO specimens of two different bulk densities are presented in this paper.

## 2. Samples and Containers

The magnesium oxide sample had been fused and was transparent, clear, and colorless; it was supplied by the Norton Company, of Niagara Falls, Ontario, Canada. Spectrochemical and spectrographic analyses at the National Bureau of Standards indicated that the sample contained 99.90 weight percent MgO if the detected metallic impurities are assumed to be present as their highest stable oxides (table 1).

Two samples of beryllium oxide were used in the present study. BeO powder was pressed, fired, and sintered to obtain bulk densities of 2.3 g cm^−3^ and 1.6 g cm^−3^ (firing temperatures of 1,800 and 1,100 °C respectively). These two samples, whose densities were about 72 and 50 percent of the single-crystal (X-ray) value, will hereafter be referred to as BeO samples 1 and 2, respectively. Spectrochemical analyses of both samples at the Bureau indicated that they contained 99.96 percent BeO by weight (table 1). In a petrographic examination, sample 1 was found to consist of approximately isometric particles 25*μ* on an edge. BeO sample 2 was observed to be composed of needlelike particles estimated to average 10*μ* in length and 1*μ*^2^ in cross section. The surface-to-volume ratio of such a particle is the same as that for a cube with an edge of 1.5*μ*. The sintered samples of BeO used in the enthalpy measurements each consisted of two cylinders 2 cm long and 1 cm in diameter.

The samples were sealed in containers of annealed pure silver preparatory to making enthalpy measurements [1]. BeO sample 2 lost weight during the first attempts at sealing it in its container. Further study of the weight following successive heat treatment and exposure to the room atmosphere showed that at least 0.3 percent of the original sample mass was lost on heating to about 1,100 °K, but was regained by the sample after cooling in a desiccator and then standing in room air for 30 min. Tests on six specimens of this lower-density sample of BeO all showed the same hygroscopic behavior. Similar tests made with BeO sample 1 showed no detectable mass change. When BeO sample 2 was finally sealed in its container its mass was the lowest attainable by the heat treatment mentioned above. It is possible, however, that the sample was still contaminated by a small amount of water.

## 3. Enthalpy Measurements

The “drop” method and calorimeter employed in the enthalpy measurements have been described in detail in a previous publication [3]. In brief, the method used was as follows. The sample, sealed in a silver container, was suspended in a silver-core furnace until it had time to come to a constant known temperature. It was then dropped (with almost free fall) into the Bunsen ice calorimeter, which measured the heat evolved by the sample plus container in cooling to 273.15 °K. In order to account for the enthalpy of the silver container and the heat lost during the drop, a similar experiment was made with an identical empty container at the same furnace temperature. The difference between the two values of heat is a measure of the enthalpy change of the sample between 273.15 °K and the furnace temperature.

The temperature of the central portion of the furnace was measured by a strain-free platinum resistance thermometer (ice-point resistance, about 24 ohms) up to and including 873 °K, and by an annealed Pt/Pt-10 percent Rh thermocouple at higher temperatures.^5^ The thermometer and thermocouple were frequently intercompared during the heat measurements, were calibrated shortly afterwards, and were concordant with the good consistency of these two instruments over a period of more than 5 years.

The measured heat values obtained in individual runs on an empty container that were used in the calculations of this paper are recorded in a previous publication [1]. The second column of table 2 gives the fully corrected measured enthalpy values, in defined thermochemical calories per mole, for the magnesium oxide. In table 3, the second and third columns contain the corresponding information for BeO samples 1 and 2. The individual enthalpy values listed in these two tables were obtained by subtracting the empty container values (using the mean at each temperature) from the observed enthalpies for sample plus container and then converting to molar values. Corrections had been applied in the usual manner [1]. The enthalpy correction for the impurities in the MgO amounted to less than 0.02 percent of the enthalpy of the sample. For BeO an impurity correction of less than 0.01 percent of the sample enthalpy was used. These corrections were made assuming that the foreign chemical elements were present as the highest stable oxides and that their enthalpies contributed additively to the total observed enthalpy. The calculated values of enthalpy in tables 2 and 3 are smoothed values arrived at as described in section 4.

## 4. Smoothed Thermodynamic Functions

The mean enthalpy values for MgO and for BeO sample 1 were used to derive eqs (1) and (2) (in cal mole^−1^ at *T* °K) by the method of least squares. The results for BeO sample 2 were ignored, for the reasons discussed in section 5.

$$\begin{array}{c} \text{MgO}:H_{T}^{{}^{\circ}}-H_{273.15}^{{}^{\circ}}=10.7409T+1.2177\left(10^{-3}\right)T^{2}\\ -2.3183\left(10^{-7}\right)T^{3}+2.26151\left(10^{5}\right)T^{-1}-3847.94. \end{array}$$ (1)

$$\begin{array}{c} \text{BeO}:H_{T}^{{}^{\circ}}-H_{273.15}^{{}^{\circ}}=11.1084T+7.1245\left(10^{-4}\right)T^{2}\\ +8.40705\left(10^{5}\right)T^{-1}-5.31245\left(10^{7}\right)T^{-2}-5453.21. \end{array}$$ (2)

Enthalpy values calculated from eqs (1) and (2) are tabulated and compared with experimental values in the last two columns of tables 2 and 3. Tables 4 and 5, which give the common thermodynamic properties of magnesium oxide and beryllium oxide, were obtained by a four-point numerical integration, at 10-deg intervals, of heat-capacity values chosen as follows. The values of heal capacity for MgO at and above 340 °K were obtained from eq (1); below 340 °K, values from low-temperature measurements [5, 6] were smoothed so as to join the derivative of eq (1) at this temperature. Equation (2) was used to obtain heat-capacity values for BeO at and above 410 °K; below 410 °K the heat capacities obtained from recent low-temperature work [6] in the room-temperature region were used in the integration. The mathematical work was performed on an IBM 7090 computer.

The thermodynamic properties of MgO in table 4 (other than *C_p_*), which are based on values for 298.15 °K obtained by smoothing low-temperature data [5, 6], are given relative to 0 °K. Because no low-temperature thermodynamic data have been published for macrocrystalline BeO, the same thermodynamic properties of this substance are given relative to 298.15 °K.

On the basis of previous work with the Bunsen ice calorimeter, the authors believe that the uncertainty of the thermodynamic properties in tables 4 and 5 corresponds to 0.4 percent in the heat capacity. Changes in values of the thermodynamic properties introduced by smooth-joining to low-temperature data are within the experimental uncertainty.

## 5. Discussion

The statements made in an earlier paper [1] concerning the usual precision of enthalpy measurements made with the Bunsen ice calorimeter apply again in the present paper. The deviation of eq (1) from the mean observed enthalpies of MgO averages 0.04 percent or 1.9 cal/mole. The corresponding numbers for eq (2) and BeO sample 1 are 0.02 percent or 0.7 cal/mole. The maximum deviation of eq (1) from the mean observed enthalpies is 0.09 percent, while that of eq (2) is 0.06 percent.

Beryllium oxide is at present difficult to obtain in the form of particles large enough to be sure that the total surface area is too small to affect the heat capacity appreciably. For this reason two samples of widely different surface-to-volume ratios were measured in this investigation in an effort to test this possible complication, which has been discussed by one of the authors in a recent publication [7]. Columns 2 and 3 of table 3 show that sample 2 consistently exhibited slightly higher relative-enthalpy values at 323 °K and 373 °K than did sample 1, though there is no consistent difference in sign at the higher temperatures. In contrast to sample 1, sample 2 had a surface-to-volume ratio 15 to 20 times as large, and also may have retained a very small amount of water (see sec. 2). Both factors would tend to increase the measured heat capacity somewhat, and may have contributed to the differences between the two samples noted up to 373 °K. However, since these differences do not exceed 1 percent, it appears unlikely that the relatively small surface area of sample 1 could in this temperature range have contributed more than a few hundredths of 1 percent to its heat capacity. All the smoothed results for beryllium oxide in this paper are based on sample 1.

**Figure 1:**
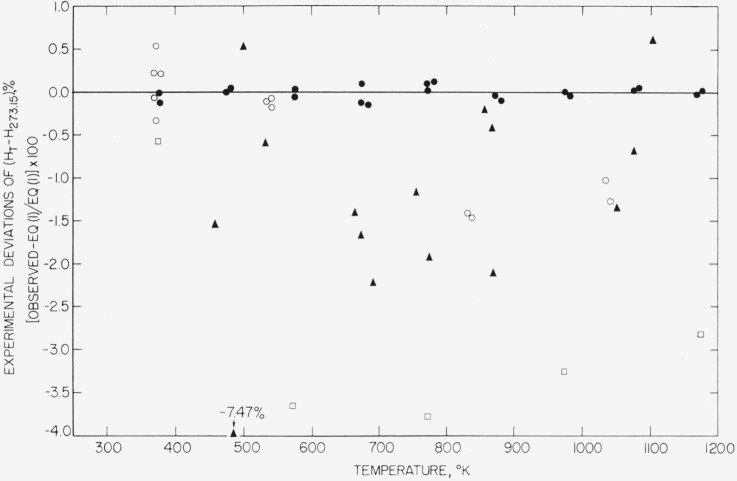
Comparison of the enthalpy, relative to 273 °K, of magnesium oxide obtained from eq (1) with individual values obtained in various investigations. (Some of the observed points have been displaced horizontally by small amounts in order to avoid the confusion of overlapping.) —, NBS smoothed, eq (1); ●, NBS observed (present paper); ▲, Arthur [8]; o, Magnus [9]; □, Wilkes [10].

Figure 1 affords a comparison of the present measurements on MgO with previously reported values [8, 9, 10] in the form of percentage deviation of individual unsmoothed measurements of $\left(H_{T}^{{}^{\circ}}-H_{273.15^{{}^{\circ}}\text{K}}^{{}^{\circ}}\right)$ from eq (1). The deviations of other workers’ values from the measurements reported in this paper are in general many times the estimated uncertainties of the authors’ values, a fact which is in line with the belief that the latter are the most accurate available for this substance.

**Figure 2:**
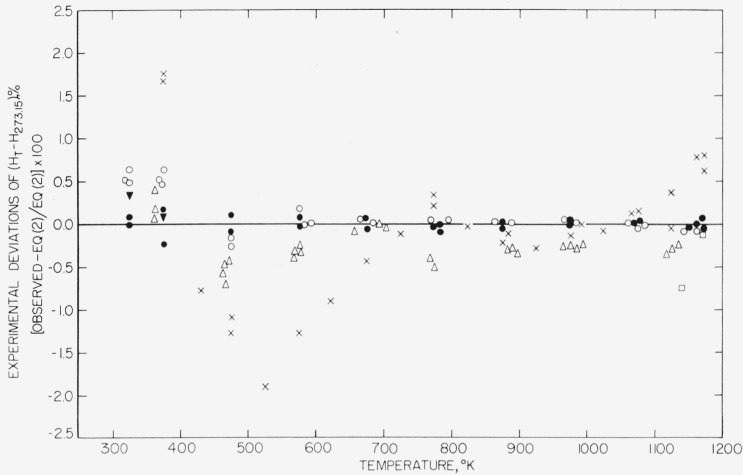
Comparison of the enthalpy, relative to 278 °K, of beryllium oxide obtained from eq (2) with individual values observed in various investigations. (Some of the observed points have been displaced horizontally by small amounts in order to avoid the confusion of overlapping.) —, NBS smoothed, eq (2); ●. NBS observed (present paper), sample 1 (“high density”); o, NBS observed (present paper), sample 2 (“low density”); x, Magnus and Danz [11]; △, Rodigina and Gomel’skii [12]; □, Kandyba et al. [13]; ▼, Furukawa and Reilly [6].

In figure 2 the present BeO measurements are compared with those of earlier workers [6, 11, 12, 13]. Of particular importance is the recent work by Rodigina and Gomel’skii on a sintered specimen, which shows a reproducibility comparable with that of the present measurements and agreement with eq (2) which is generally better than 0.5 percent of the enthalpy relative to 273.15 °K.

Magnesium oxide is probably the more favorable of the two materials as a high-temperature heat-capacity standard, since it is available in large crystals of reasonably high purity. The agreement between BeO samples 1 and 2 at and above 473 °K and the unusually good agreement with one other worker [12] on a specimen similar to sample 1 support the serious consideration of sintered beryllium oxide as a heat-capacity standard, subject to the conditions that the samples used do not have too small a particle size and are not contaminated by adsorbed water.

A phase change in BeO has reportedly been observed at approximately 2,220 °K [14]. Such a transition would adversely affect the use of beryllium oxide as a heat-capacity standard at higher temperatures. Measurements on this material up to 1,800 °K or higher are planned at the National Bureau of Standards to extend the temperature range of available accurate values. At the present time the heat-capacity discrepancy between reference [13] and the present work is greater than 2.5 percent at 1,200 °K.

## Figures and Tables

**Table 1:** Impurities in the samples

Element^a^	BeO sample 1	MgO
		
	*Weight %*	*Weight %*
Ag	(c)	<0.001
Al	0.007	.004
Be	(b)	(c)
Ca	<.001	.025
Cr	(c)	<.001
Cs	.001	(c)
Cu	<.001	<.001
Fe	.001	.02
K	.002	(c)
Li	<.00005	(c)
Mg	<.00005	(b)
Mn	(c)	.008
Na	.002	<.002
Si	.01	.009

a The samples were also examined for the following elements which were not detected: As, Au, B, Ba, Bi, Cd, Ce, Co, Ga, Ge, Hf, In, Ir, La, Mo, Nb, Ni, Os, P, Pb, Pd, Pt, Rh, Ru, Sb, Sc, Sn, Sr, Ta, Te, Th, Ti, U, V, W, Y, Zn, Zr. In addition, the following elements were undetected in BeO: Dy, Er, Eu, Gd, Ho, Lu, Nd, Pr, Ra, Rb, Re, Sm, Tb, Tm, Yb.

b Major constituent.

c Not detected.

**Table 2:** Relative enthalpy of magnesium oxide^a^ (*H_T_*−*H*_273.15_°_K_)

Furnace temperature, *T*	Individual enthalpy measurements^b^	Mean observed enthalpy	Calc. eq (1)	Mean observed–calc.
				
°*K*	*cal mole*^−1^	*cal mole*^−1^	*cal mole*^−1^	*cal mole*^−1^
373.15	$\left\{ \begin{array}{l} 923.5\\ 922.7 \end{array}\right.$	} 923.1	923.6	−0.5
473.15	$\left\{ \begin{array}{l} 1960.3\\ 1961.1 \end{array}\right.$	} 1960.7	1960.1	+0.6
573.15	$\left\{ \begin{array}{l} 3060.4\\ 3058.0 \end{array}\right.$	} 3059.2	3059.2	+0.0
673.15	$\left\{ \begin{array}{l} 4194.8\\ 4203.6\\ 4194.0 \end{array}\right.$	} 4197.5	4199.3	−1.8
773.15	$\left\{ \begin{array}{l} 5375.5\\ 5371.0\\ 5376.7 \end{array}\right.$	} 5374.4	5369.6	+4.8
873.15	$\left\{ \begin{array}{l} 6560.6\\ 6558.2 \end{array}\right.$	} 6559.4	6563.5	−4.1
973.15	$\left\{ \begin{array}{l} 7777.6\\ 7773.6 \end{array}\right.$	} 7775.6	7776.5	−0.9
1073.15	$\left\{ \begin{array}{l} 9007.5\\ 9009.1 \end{array}\right.$	} 9008.3	9005.3	+3.0
1173.15	$\left\{ \begin{array}{l} 10245.4\\ 10246.6 \end{array}\right.$	} 10246.0	10247.1	−1.1

a Mol wt = 40.311 g.

b Sample mass = 10.7578 g.

**Table 3:** Relative enthalpy of beryllium oxide^a^ (*H_T_*−*H*_273.15_°_K_)

Furnace temperature, *T*	Individual enthalpy measurements		Mean observed enthalpy (sample 1)	Calc. eq (2)	Mean observed–calc.
	Sample 1^b^	Sample 2^c^			
					
°*K*	*cal mole*^−1^	*cal mole*^−1^	*cal mole*^−1^	*cal mole*^−1^	*cal mole*^−1^
323.15	$\left\{ \begin{array}{l} 303.6\\ 303.9 \end{array}\right.$	305.6 305.5	} 303.7	303.7	+0.0
373.15	$\left\{ \begin{array}{l} 660.9\\ 663.6\\ \ldots \ldots \ldots . \end{array}\right.$	665.5 665.8 666.6	} 662.2	662.6	−0.4
473.15	$\left\{ \begin{array}{l} 1503.1\\ 1500.4 \end{array}\right.$	1499.3 1497.8	} 1501.8	1501.8	−0.0
573.15	$\left\{ \begin{array}{l} 2455.1\\ 2452.3\\ \ldots \ldots \ldots . \end{array}\right.$	2457.4 2452.6 2453.3	} 2453.7	2452.7	+1.0
673.15	$\left\{ \begin{array}{l} 3481.6\\ 3477.0 \end{array}\right.$	3479.8 3481.0	} 3479.3	3478.9	+0.4
773.15	$\left\{ \begin{array}{l} 4558.0\\ 4555.1\\ 4560.3 \end{array}\right.$	4562.2 4562.9 ………	} 4557.8	4559.6	−2.2
873.15	$\left\{ \begin{array}{l} 5679.8\\ 5684.1 \end{array}\right.$	5684.0 5684.2	} 5682.0	5682.4	−0.4
973.15	$\left\{ \begin{array}{l} 6843.4\\ 6838.1 \end{array}\right.$	6841.4 6843.3	} 6840.7	6839.5	+1.2
1073.15	$\left\{ \begin{array}{l} 8025.9\\ 8026.8\\ \ldots \ldots \ldots . \end{array}\right.$	8026.7 8022.2 8024.2	} 8026.3	8025.6	+0.7
1173.15	$\left\{ \begin{array}{l} 9242.4\\ 9232.6\\ 9237.6\\ 9233.6 \end{array}\right.$	……… 9228.5 9228.7 ………	} 9236.5	9237.2	−0.7

a Mol wt = 25.012 g.

b Mass of sample 1 = 6.7171 g.

c Mass of sample 2 = 6.3747 g.

**Table 4:** Thermodynamic properties of magnesium oxide

*T*	$C_{p}^{{}^{\circ}}$	$H_{T}^{{}^{\circ}}-H_{0^{{}^{\circ}}K}^{{}^{\circ}}$	$S_{T}^{{}^{\circ}}-S_{0^{{}^{\circ}}K}^{{}^{\circ}}$	$-\frac{F_{T}^{{}^{\circ}}-H_{0^{{}^{\circ}}K}^{{}^{\circ}}}{T}$
				
*°K*	*cal/mole-deg*	*cal*/*mole*	*cal/mole-deg*	*cal/mole-deg*
298.15	8.906	1234.6	6.430	2.298
300	8.939	1251.1	6.494	2.324
320	9.261	1433.2	7.082	2.603
340	9.532	1621.2	7.651	2.883
360	9.782	1814.4	8.204	3.164
380	10.000	2012.3	8.738	3.443
400	10.190	2214.2	9.256	3.721
420	10.359	2419.7	9.758	3.996
440	10.510	2628.4	10.243	4.269
460	10.645	2840.0	10.713	4.539
480	10.768	3054.2	11.169	4.806
500	10.880	3270.6	11.611	5.070
550	11.122	3820.9	12.660	5.712
600	11.324	4382.2	13.636	6.332
650	11.495	4952.8	14.549	6.930
700	11.643	5531.3	15,407	7.505
750	11.774	6116.8	16.215	8.059
800	11.891	6708.5	16.978	8.593
850	11.996	7305.7	17.702	9.108
900	12.090	7907.8	18.391	9.604
950	12.176	8514.6	19.047	10.084
1000	12.255	9125.4	19.673	10.548
1050	12.326	9739.9	20.273	10.997
1100	12.391	10358	20.848	11.432
1150	12.451	10979	21.400	11.853
1200	12.505	11603	21.931	12.262

**Table 5:** Thermodynamic properties of beryllium oxide

*T*	$C_{p}^{{}^{\circ}}$	$H_{T}^{{}^{\circ}}-H_{298.15^{{}^{\circ}}\text{K}}^{{}^{\circ}}$	$S_{T}^{{}^{\circ}}-S_{298.15^{{}^{\circ}}\text{K}}^{{}^{\circ}}$	$-(\frac{F_{T}^{{}^{\circ}}-H_{298.15^{{}^{\circ}}\text{K}}^{{}^{\circ}}}{T})-S_{298.15^{{}^{\circ}}\text{K}}^{{}^{\circ}}$
				
°*K*	*cal/mole-deg*	*cal/mole*	*cal/mole-deg*	*cal/mole-deg*
298.15	6.102	0	0	0
300	6.146	11.3	0.038	0.000
320	6.597	138.8	.449	.015
340	7.012	275.0	.862	.053
360	7.393	419.1	1.273	.109
380	7.746	570.5	1.683	.181
400	8.078	728.8	2.089	.267
420	8.375	893.4	2.490	.363
440	8.640	1063.6	2.886	.469
460	8.882	1238.8	3.275	.582
480	9.104	1418.7	3.658	.702
500	9.308	1602.9	4.034	.828
550	9.752	2079.7	4.943	1.161
600	10.120	2576.8	5.807	1.513
650	10.432	3090.8	6.630	1.875
700	10.700	3619.2	7.413	2.243
750	10.934	4160.2	8.160	2.613
800	11.142	4712.2	8.872	2.982
850	11.329	5274.0	9.553	3.348
900	11.499	5844.8	10.206	3.711
950	11.654	6423.7	10.831	4.070
1000	11.799	7010.1	11.433	4.423
1050	11.934	7603.4	12.012	4.771
1100	12.061	8203.3	12.570	5.112
1150	12.181	8809.4	13.109	5.449
1200	12.296	9421.4	13.630	5.779

## Footnotes

4 Figures in brackets indicate the literature references at the end of this paper.

5 Temperatures are on the International Temperature Scale of 1948 as modified in 1954 [4].
